# Interplay between SARS‐CoV‐2 and human long non‐coding RNAs

**DOI:** 10.1111/jcmm.16596

**Published:** 2021-05-09

**Authors:** Maryam Moazzam‐Jazi, Hossein Lanjanian, Samaneh Maleknia, Mehdi Hedayati, Maryam S. Daneshpour

**Affiliations:** ^1^ Cellular and Molecular Endocrine Research Center Research Institute for Endocrine Sciences Shahid Beheshti University of Medical Sciences Tehran Iran; ^2^ Basic and Molecular Epidemiology of Gastrointestinal Disorders Research Center, Research Institute for Gastroenterology and Liver Diseases Shahid Beheshti University of Medical Sciences Tehran Iran

**Keywords:** Cis regulation, COVID‐19, long non‐coding RNA, SARS‐CoV‐2 genome, trans regulation

## Abstract

The long non‐coding RNAs (lncRNAs) play a critical regulatory role in the host response to the viral infection. However, little is understood about the transcriptome architecture, especially lncRNAs pattern during the SARS‐CoV‐2 infection. In the present study, using publicly available RNA sequencing data of bronchoalveolar lavage fluid (BALF) and peripheral blood mononuclear cells (PBMC) samples from COVID‐19 patients and healthy individuals, three interesting findings highlighted: (a) More than half of the interactions between lncRNAs‐PCGs of BALF samples established by three trans‐acting lncRNAs (HOTAIRM1, PVT1 and AL392172.1), which also exhibited the high affinity for binding to the SARS‐CoV‐2 genome, suggesting the major regulatory role of these lncRNAs during the SARS‐CoV‐2 infection. (b) lncRNAs of MALAT1 and NEAT1 are possibly contributed to the inflammation development in the SARS‐CoV‐2 infected cells. (c) In contrast to the 3′ part of the SARS‐CoV‐2 genome, the 5′ part can interact with many human lncRNAs. Therefore, the mRNA‐based vaccines will not show any side effects because of the off‐label interactions with the human lncRNAs. Overall, the putative functionalities of lncRNAs can be promising to design the non‐coding RNA‐based drugs and to inspect the efficiency of vaccines to overcome the current pandemic.

## 1 INTRODUCTION

The recent outbreak of COVID‐19 has been leading to an increased number of infected individuals and subsequent mortality worldwide. Although genomic variants can cause differences in the symptoms and contagion of SARS‐CoV‐2 infection,^1^ it is essential to evaluate the various aspects of viral pathogenesis to understand the biological pathways relevant to the COVID‐19 pandemic. Long non‐coding RNAs (lncRNAs), RNA molecules longer than 200 nucleotides, can serve as diagnostic biomarkers or therapeutic targets for many diseases. Usually, lncRNAs can activate or silence gene expression locally via the cis‐acting manner on the proximal protein‐coding genes or globally through the trans‐acting manner on the distant genes. In the cytoplasm, lncRNAs can interact with target mRNAs through base‐pairing to either stabilize mRNAs and enhance or inhibit their translation.^2^ The role of lncRNAs in the viral infection, including the initiation and progression of infectious diseases, has been recently reported. RNA sequencing of SARS‐CoV‐ and influenza A‐infected lung tissues of mice also demonstrated the key roles of lncRNAs in respiratory virus pathogenesis via stimulating the interferon (IFN) production.^3^ In our recent work, we found that the miR‐29 family has the most binding sites (11 sites) on the SARS‐CoV‐2 genome.^4^ However, to our knowledge, there is not any report on investigating the physical interaction of human differentially expressed lncRNAs with SARS‐CoV‐2. In the present study, using the available transcriptomic data obtained from the peripheral blood mononuclear cells (PBMC) and bronchoalveolar lavage fluid (BALF) samples of COVID‐19 patients and healthy individuals, we focussed on the cis‐ and trans‐acting differentially expressed (DE) lncRNAs and their potential functions in response to the virus infection. Furthermore, we surveyed the potential interaction of DE lncRNAs with the SARS‐CoV‐2 genome.

## 2 MATERIALS AND METHODS

### 2.1 Data collection and processing

The raw RNA sequencing data of 12 Chinese individuals (PBMC and BALF) deposited at the Beijing Institute of Genomics (BIG) Data Center (accession number: CRA002390) was used in the present study.^5^ After checking the read quality and trimming, reads were mapped to the human genome (hg38) using STAR (V. 2.7.2b) with the ENCODE standard options​.^6^ Then, the count matrix was generated, and differentially expressed genes were identified using edgeR package (V.3.7).^7^ The genes with a read count greater than 15 were chosen and normalized to counts per millions (CPM). For the BALF data analysis, we summed up the read counts from the two technical replicates of COVID‐19 patients to create an object with a single column of reading count for each patient sample. Here, genes with log_2_ fold change > |1| and false discovery rate (FDR) threshold of 0.05 considered significantly differentially expressed for further analysis. Genes with the biotypes of processed_transcript, pseudogene, lincRNA, 3 prim_overlapping_ncrna, antisense, sense_intronic and sense_overlapping were considered as lncRNAs for further analysis.

### 2.2 Identification of cis‐acting lncRNAs

The lncRNAs located at the adjacent (300 kbp upstream and downstream) of protein‐coding genes (PCGs) are considered as cis‐acting lncRNAs if they exhibited a high correlation expression with the adjacent PCGs (correlation coefficient >.95 or <−.95 at the adjusted *P*‐value cut‐off of .05). The correlation coefficient between DE lncRNAs and DE PCGs calculated using the Hmisc package implemented in R. Spearman's rank correlation test was utilized for doing this analysis.

### 2.3 Identification of trans‐acting lncRNAs

We screened the trans‐acting lncRNAs by comparing the complementary bases between PCGs and lncRNAs using the LncTar tool.^8^ Here, PCGs and lncRNAs with high fold change threshold (log_2_FC cut off of |2|) were utilized to ensure the possibility or impossibility for the physical interaction between the lncRNAs and the target genes. Additionally, we investigated the possible interaction of DE lncRNAs with the complete genome sequence of SARS‐CoV‐2 (GenBank: MN988668) by the LncTar tool.

### 2.4 Functional annotation of lncRNAs

The biological function of DE lncRNAs was identified by gene set enrichment analysis of DE PCG targets of lncRNAs using the g:Profiler tool.^9^ The Go terms or biological pathways with FDR < 0.05 were considered significant.

## 3 RESULTS AND DISCUSSION

We detected 207 and 223 lncRNAs as significantly altered genes in BALF and PMBC samples, respectively (File S1). LincRNA and antisense were the main classes of differentially expressed lncRNAs in both PBMC and BALF samples. Among the dysregulated lncRNAs, 17% of lncRNAs in PBMC samples and about 50% in BALF samples were up‐regulated.

### 3.1 Identification of DE cis‐acting lncRNAs in response to the SARS‐CoV‐2 infection

We found that the expression of 239 and 527 PCGs at the PBMC and BALF samples could be influenced by 106 and 162 cis‐acting lncRNAs, respectively. Based on our enrichment results, these lncRNAs mainly play a role in the immune‐related processes in the PBMC samples. The GO terms, like immune system process, myeloid leukocyte activation, neutrophil degranulation and the regulation of ion homeostasis were significantly associated with this type of RNA molecules during the SARS‐CoV‐2 infection (File S2). Specifically, nine cis DE lncRNAs were highly correlated (correlation coefficient >.9 or <−.9, adjusted *P*‐value <.05) with the known genes involved in the immune system (Table 1). All cis DE lncRNAs except for AC009088 were positively correlated with immune‐related genes, suggesting their function as the potential transcriptional enhancer of the neighbouring protein‐coding genes and implying the role of cis lncRNAs in the immune system behaviour. AC009088 is a kind of antisense lncRNA transcribed from the opposite strand of Pycard gene.^10^ The Pycard up‐regulation accompanied the reduced expression of this lncRNA in SARS‐CoV‐2‐infected samples (PMBC) compared to control, suggesting the potential transcriptional inhibitory function of this lncRNA, which could be used for fine‐tuning the inflammatory processes and therapeutic purposes. Pycard is one of the key components of the NLRP3 inflammasome that contributed to hyper‐inflammation and disease severity during Influenza (IAV) infection, which its therapeutic suppression can be one of the treatment opportunities for this disease.^11^ The transcript level of NEAT1 and MALAT1 was significantly up‐regulated in the patient's BALF samples compared to healthy samples, which is in line with a recent report on SARS‐CoV‐2‐infected human bronchial epithelial cells.^12^ The increased expression level of these lncRNAs also determined in the PBMC sample of severe COVID‐19 patients compared to moderate patients and healthy individuals.^13^ These known lncRNAs (MALAT1 and NEAT1) are possibly contributed to the inflammation development in the SARS‐CoV‐2 infected cells. Our further investigation revealed that both lncRNAs were negatively correlated with CAPN1 (Table 1), a cysteine protease involved in the influenza virus infection.

**TABLE 1:** Significant correlation of DE cis long non‐coding RNAs with DE protein‐coding genes related to the immune system within PBMC and BALF samples

Sample	Protein‐coding gene	Long non‐coding RNA	*r* ^2^	Adjusted *P*‐value
PBMC	PYCARD	AC009088.1	−.95	.023
	RNF135	AC138207.9	1	.0001
	CD247	AL359962.3	.942	.023
	CTSD	AC068580.3	.942	.023
	CTSD	AC068580.1	.942	.023
	IFN‐γ	LINC02384	.942	.023
	RPS6KA5	AL135818.3	.942	.023
	RNF135	AC138207.4	1	.000
	CEBPA	CEBPA‐DT	.942	.023
BALF	CTSD	AC068580.3	1	.001
	CAPN1	NEAT1	−.98	.001
	CAPN1	MALAT1	−.98	.0012
	TSPAN32	KCNQ1OT1	−1	.002
	CD81	KCNQ1OT1	−1	.0031

### 3.2 Identification of DE trans‐acting lncRNAs in response to the SARS‐CoV‐2 infection

**FIGURE 1:**
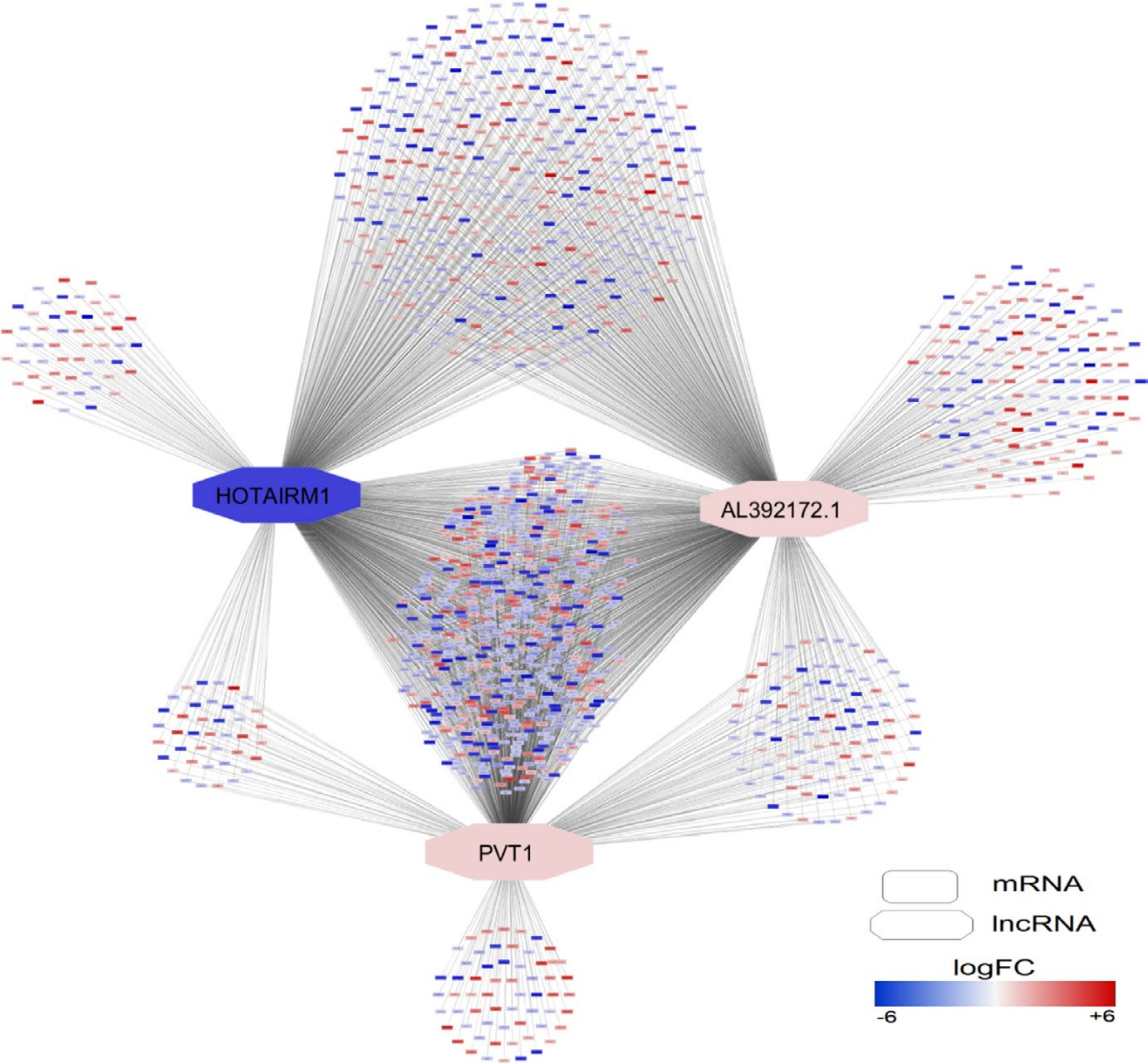
Biological network of trans‐acting lncRNAs interacted with protein‐coding genes within BALF samples. The network was drawn using Cytoscape tool (version 3.8) available at https://cytoscape.org/

According to our results, 37 differentially expressed trans‐lncRNAs had the potential binding site on 1603 differentially expressed protein‐coding genes in the BALF sample. Interestingly, we found that 68% of interactions between lncRNAs and PCGs were covered by three trans‐lncRNAs named AL392172, HOTAIRM1 and PVT1 (Figure 1), implying their principal roles in regulating the corresponding differentially expressed PCGs during SARS‐CoV‐2 infection. These trans‐acting lncRNAs were significantly related to multiple GO terms and biological pathways, including structural constituent of ribosome, chemokine activity, chemokine receptor binding, viral transcription, cytokine‐cytokine receptor interaction, IL‐17 signalling pathway and Nonsense‐Mediated Decay (NMD) pathway (File S3). Similarly, we recognized the 112 trans‐acting lncRNAs regulating the expression of 169 target protein‐coding genes within the PBMC samples. Interestingly and unlike the cis‐acting lncRNAs, the enrichment analysis indicated that almost all PBMC trans‐acting lncRNAs were relevant to the cell cycle processes. GO terms and pathways related to cell cycle processes, including cell division, cell cycle regulation, cell cycle phase transition and cyclin A/B1/B2 associated events, were significantly enriched and can be modulated by these lncRNAs during the SARS‐CoV‐2 infection (File S4). We also detected multiple genes and pathways relevant to DNA damage and apoptosis processes that can be regulated via the trans‐lncRNAs of PBMC. It may suggest that SARS‐CoV‐2, similar to the infectious bronchitis virus (IBV) and SARS‐CoV, induce cell cycle arrest and apoptosis via the activation of a DNA damage pathways to facilitate viral replication.^14^

### 3.3 The DE lncRNAs interaction with the SARS‐CoV‐2 genome

With the dnG score of less than −8, a very stringent cut‐off, 20 DE lncRNAs of PBMC samples could bind to the different viral genomic positions (File S5). Surprisingly, the start binding position of all lncRNAs was the first viral genome nucleotide that spanned at least to the 6221th nucleotide of the genome. This interval encompasses the part of the ORF1ab gene that encodes the NSP1, NSP2 and NSP3. In the same perspective, 56 DE lncRNAs of BALF samples exhibited the capability of binding to the SARS‐CoV‐2 genome, which most of them covered 6083‐13487 nucleotide in length started from the first viral genome nucleotide (File S6). Besides establishing most interactions between DE PCG‐trans‐lncRNA, PVT1 and HOTAIRM1 were showed a high affinity for binding to the virus genome. The human lncRNA‐virus genome interaction sites appear to restrict the ORF1ab gene and rarely span NSP5 or NSP6. Consistent with our findings, Vandelli et al demonstrated the 5′ end of the viral genome is highly structured and can interact with various human proteins.^15^ Also, the viral portion of SARS‐CoV‐2 harbouring the sequence coding spike protein tends to interact neither with human proteins nor with human lncRNAs, implying that the mRNA‐based vaccines will not show the possible side effects because of the off‐label interactions with these macromolecules.

## CONFLICT OF INTEREST

The authors confirm that there are no conflicts of interest.

## AUTHOR CONTRIBUTIONS

**Maryam Moazzam‐Jazi:** Conceptualization (equal); Data curation (equal); Formal analysis (equal); Investigation (equal); Methodology (equal); Resources (equal); Software (equal); Writing‐original draft (equal). **Hossein Lanjanian:** Conceptualization (equal); Data curation (equal); Formal analysis (equal); Investigation (equal); Software (equal). **Samaneh Maleknia:** Formal analysis (equal); Software (equal). **Mehdi Hedayati:** Conceptualization (equal); Funding acquisition (equal); Supervision (equal). **maryam alsadat daneshpour:** Conceptualization (lead); Supervision (lead).

## Supporting information

App S1

## Data Availability

The data that support the findings of this study are available in the Genome Sequence Archive of Beijing Institute of Genomics (BIG) Data Center at https://bigd.big.ac.cn/ with the reference number of CRA002390.
